# Expression of the Human Glucokinase Gene: Important Roles of the 5′ Flanking and Intron 1 Sequences

**DOI:** 10.1371/journal.pone.0045824

**Published:** 2012-09-20

**Authors:** Yi Wang, Tingting Guo, Shuyong Zhao, Zhixin Li, Yiqing Mao, Hui Li, Xi Wang, Rong Wang, Wei Xu, Rongjing Song, Ling Jin, Xiuli Li, David M. Irwin, Gang Niu, Huanran Tan

**Affiliations:** 1 Department of Pharmacology, Peking University, Health Science Center, Beijing, China; 2 Department of Pharmacology, Chifeng College, Chifeng, China; 3 Department of Laboratory Medicine and Pathobiology, University of Toronto, Toronto, Ontario, Canada; 4 Beijing N&N Genetech Company, Beijing, China; University of Texas Health Science Center/South Texas Veterans Health Care System, United States of America

## Abstract

**Background:**

Glucokinase plays important tissue-specific roles in human physiology, where it acts as a sensor of blood glucose levels in the pancreas, and a few other cells of the gut and brain, and as the rate-limiting step in glucose metabolism in the liver. Liver-specific expression is driven by one of the two tissue-specific promoters, and has an absolute requirement for insulin. The sequences that mediate regulation by insulin are incompletely understood.

**Methodology/Principal Findings:**

To better understand the liver-specific expression of the human glucokinase gene we compared the structures of this gene from diverse mammals. Much of the sequence located between the 5′ pancreatic beta-cell-specific and downstream liver-specific promoters of the glucokinase genes is composed of repetitive DNA elements that were inserted in parallel on different mammalian lineages. The transcriptional activity of the liver-specific promoter 5′ flanking sequences were tested with and without downstream intronic sequences in two human liver cells lines, HepG2 and L-02. While glucokinase liver-specific 5′ flanking sequences support expression in liver cell lines, a sequence located about 2000 bases 3′ to the liver-specific mRNA start site represses gene expression. Enhanced reporter gene expression was observed in both cell lines when cells were treated with fetal calf serum, but only in the L-02 cells was expression enhanced by insulin.

**Conclusions/Significance:**

Our results suggest that the normal liver L-02 cell line may be a better model to understand the regulation of the liver-specific expression of the human glucokinase gene. Our results also suggest that sequences downstream of the liver-specific mRNA start site have important roles in the regulation of liver-specific glucokinase gene expression.

## Introduction

Mutations in the human glucokinase (GCK) gene can lead to the MODY2 form of diabetes [1] and to persistent hyperinsulinemic hypoglycemia of infancy (PHHI) [2]. Inactivating mutations cause MODY2 while activating mutations result in PHHI [1], [2]. Glucokinase, the enzyme encoded by the GCK gene, catalyzes the reaction ATP + D-glucose → ADP + D-glucose-6-phosphate, the first and obligatory step for glucose utilization after glucose is transported into a cell, and allows the storage of excess glucose as glycogen in liver cells [3]. Phosphorylation of glucose not only is a key step in the metabolism of glucose but also involved in the sensing of blood glucose levels, thereby regulating the release of insulin by pancreatic beta cells [3], [4]. Glucokinase, thus, has important roles both in regulating insulin release from pancreatic beta-cells and metabolism of glucose in liver cells, both of which have significant impact on blood glucose levels. Deficiency of glucokinase from either the pancreas or the liver has been shown to have important roles in the development of MODY2 [5].

The structure of the glucokinase genes has been characterized in human, mouse and rat [6]–[8] where it has been shown to have two promoters that are expressed in a tissue-specific pattern [4], [9]. The 5′ promoter is used by pancreatic beta-cells, as well as a few cells from the gut and central nervous system, and in human and rodent glucokinase genes is located 25–30 kb upstream of a 3′ promoter that is used exclusively by liver cells [4], [9]. While the sequences and protein factors necessary for expression of the glucokinase promoter in beta-cells has been characterized, with only 294 bases of 5′ flanking sequence required for tissue-specific expression in transgenic mice [10], [11], our knowledge of the sequences and protein factors essential for liver-specific promoter activity is incomplete [4], [9]. Transgenic studies have indicated that more than 7.5 kb of liver-specific glucokinase promoter 5′ flanking sequence are required for liver-specific expression [7], [12]. Transgenic mice bearing 83 kb of the human glucokinase locus do show both liver-specific and beta-cell specific expression, with the liver-specific expression regulated by insulin, thus appearing to contain the entire glucokinase locus [12]. Liver-specific expression of glucokinase is absolutely dependent upon insulin [4], [9], [13]. While the PI3-kinase and Protein kinase B (PKB, also called Akt) are known to be necessary for the regulation of liver-specific glucokinase expression [14], the specific factors that interact with the glucokinase gene remain elusive [4]. Some studies have suggested a role for SREBF (SREBP-1c) [15], [16] however other experiments have failed to show a direct role of SREBF including the failure to find SREBF binding to the glucokinase liver-specific promoter [17], [18]. Similarly, contradictory evidence has been advanced for a role by FOXO1, where little change in glucokinase levels was seen in the livers of Foxo1 knockout mice [19] but in vitro studies suggest that Foxo1 inhibits HNF4-potentiated expression [20]. Most functional studies have focused on the promoter and 5′ flanking regions of the liver-specific glucokinase promoter. Early DNase I hypersensitive site mapping studies indicated that hypersensitive sites, which are potential regulatory regions, exist not only in the 5′ flanking sequence, but also exist in the 1^st^ intron downstream of the liver-specific 1^st^ exon of the glucokinase gene [7], [21], [22]. Here we examined sequences near the human liver-specific glucokinase promoter and demonstrate that intron 1 sequence play a role in the expression of the human glucokinase gene in human liver cells.

## Materials and Methods

### Bioinformatic Analysis

The genomic sequence encoding the human glucokinase gene was downloaded from Release 65 of the Ensembl database (http://www.ensembl.org) in December 2011. Additional mammalian glucokinase gene sequences were identified from the Ensembl database, release 65, either due to their annotation as orthologs of the human GCK gene or by searching the genome sequences using BLAST with the human glucokinase protein sequence. Repetitive DNA elements in the genomic sequences were identified using RepeatMasker [23] (http://www.repeatmasker.org). Genomic sequences were aligned using the program MultiPipMaker [24], [25]. Potential transcription factor binding sites in the human glucokinase genomic sequences were identified using Matinspector (http://www.genomatix.de) and TFSEARCH (http://www.cbrc.jp/research/db/TFSEARCH.html).

### Plasmid Constructs

Human glucokinase genomic sequences were amplified using the polymerase chain reaction from human genomic DNA purchased from Clontech (Palo Alto, USA). Primers used to make the reporter gene constructs (see Fig. S1) are listed in Table S1. Glucokinase liver-specific 5′ flanking sequences spanning bases −3,815 to +135 and base −1,049 to +135, relative to the liver-specific mRNA start site, were amplified from genomic DNA with primers that had HindIII and SacI restriction endonuclease digestion sites added, to the 5′ and 3′ primers, respectively, to aid in directional cloning. Genomic fragments were cloned into pMD18-T (Takara, Japan) generating plasmids pGK-3815T and pGK-1049T and the sequences were verified by sequencing. These clones were then used to construct the reporter gene plasmids. The 3950 bp and 1,184 bp HindIII-SacI genomic fragments, containing the liver-specific promoter, were cloned into the luciferase reporter plasmid pGL2 Basic (Promega, USA) to generate pGK-3815Luc and pGK-1049Luc plasmids, respectively. Glucokinase intron 1 sequence, representing bases +211 to +4,868, relative to the mRNA start site, was amplified from genomic DNA with primers that included BamHI and SalI recognition sites at the 5′ and 3′ ends, respectively. Plasmid pGK-intr1Luc was constructed by inserting the 4,658-bp BamHI-SalI intron 1 genomic fragment into the corresponding sites of pGL2 Basic, downstream of the luciferase reporter gene transcription termination site. To generate glucokinase promoter-reporter constructs that contained intron sequences, plasmids pGK-3815T and pGK-1049T were digested with SacI and the respective 4 kb and 1 kb SacI-SacI fragments were ligated into the corresponding site of pGK-intr1Luc in the sense orientation. These ligations yielded plasmids pGK-3815intr1Luc and pGK-1049intr1Luc, reporter plasmids that contain both human glucokinase promoter and intron sequences, with the intron sequences located 3′ to the reporter gene transcription termination site, and thus they were not included in the luciferase mRNA transcript. Plasmids pGK-38Luc, pGK-161Luc, pGK-345Luc, pGK-571Luc pGK-753Luc and pGK-969Luc (see Fig. S1) were constructed by inserting 38-bp, 161-bp, 345-bp, 571-bp, 753-bp, 969-bp of liver-specific glucokinase promoter DNA together with 135 bp of exon 1 sequence, amplified by PCR, and cloned into the HindIII and SacI in pGL2 Basic. To test the activity of fragments of intron 1, 500–1000 base long sections of intron 1 (segments I-1– I-6, Fig. S1) were amplified by PCR and inserted into the BamHI-SalI sites 3′ to the luciferase reporter gene in the pGK-1049Luc reporter plasmid (see Table S1 for primers). Smaller portions of segment I-3 (a – e, see Fig. S1) were also amplified and cloned into the BamHI-SalI site downstream of the luciferase reporter gene in pGK-1049Luc reporter plasmid vector.

### Cell Culture and Transfection

The human hepatoma cell line HepG2 [26] was cultured in Dulbecco’s modified Eagle’s medium (DMEM) containing 25 mM glucose, supplemented with antibiotics and 10% fetal calf serum in 75 cm^2^ Tissue culture flasks (Corning, USA). The normal liver cell line L-02 [27] is of human origin and was purchased from the Type Culture Collection of Chinese Academy of Sciences, Shanghai. L-02 cells were cultured in RPMI-1640 media (Sigma, USA) containing 5.6 mM glucose, 10% fetal bovine serum, 100 µg/ml streptomycin sulfate, and 100 units/ml penicillin at 37°C in 5% CO2. The hamster insulinoma cell line HIT [28] was a gift from Professor Chun-Yan Zhou (Peking University). Monolayer culture of HIT-T15 was maintained as previously described [29]. Human illeocecal adenocarcinoma cells, HCT-8 [30] cells were maintained in RPMI-1640 (Sigma, USA) supplemented with 10% fetal calf serum (Life Technologies, USA), 1% penicillin/streptomicin and 1% L-glutamine (Sigma, USA). When cells reached 50–60% confluency, they were fed fresh medium and harvested by trypsinization. Cells were washed once in culture media containing 10% newborn calf serum and once in about 5 ml of ice-cold electroporation buffer, HEPES Buffered Saline containing 140 mM NaCl, 5 mM KCl, 0.75 mM Na_2_HPO_4_, 6 mM glucose, 25 mM HEPES (pH 7.2) with centrifugations at 800r/min for 5 min. The final cell pellet was resuspended in ice-cold electroporation buffer to provide a cell density of 10^6^ viable cells/ml and 0.4 ml samples of cell suspension were pipetted into electroporation cuvettes with an electrode gap of 4 mm. Plasmids were added in an amount of 20 µg pGL2 Basic or an equimolar amount of the pGK-Luc plasmids. For co-transfection, 2 µg of the Renilla vector (Promega, USA) were added, and the cuvettes were placed in ice for 5 min. Cells were carefully resuspended by manual shaking and electroporated with a GenePulser apparatus (Bio-Rad, USA). Capacitance and voltage were optimized for electroporation and a capacitance of 960 mF and voltage of 220 V was found to yield transfection efficiency of 54% and 48% for HepG2 and L-02 cells, respectively. Cuvettes were returned to ice for 10 min and the cells were then transferred to tubes containing 1.2 ml of DMEM plus antibiotics and 10% fetal calf serum and 200 µl aliquots of the cell suspension were pipetted into wells of 96-well culture plates (Corning, USA). After 3 h of culture in a CO2 incubator the medium was replaced. Glucokinase promoter reporter gene constructs were transfected in cells cultured in DMEM, for HepG2, or RPMI-1640, for L-02, media under three different conditions: (1) with insulin (100 nM), (2) with 10% fetal calf serum, and (3) with neither insulin nor serum. Cell culture was continued for 20 h and then cells were cell harvested for the measurement of firefly and Renilla luciferase activities.

### Reporter Enzyme Assays

Reporter gene assays of the transfected cells were performed using the Dual-Luciferase Reporter Assay kit (Promega, USA) and luminescence was measured using a GloMax 96 Microplate Luminometer (Promega, USA). Results were expressed as the ratio of luciferase luminescence to Renilla luminescence, thereby normalizing the results to the internal control.

### RT-PCR

Endogenous expression of GCK (glucokinase), GCKR (glucokinase regulatory protein), PCK1 (phosphoenol pyruvate kinase), GYS2 (glycogen synthase 2), PYGL (glycogen phosphorylase), G6PC (glucose-6 phosphotase) INSR (insulin receptor), SREBF (sterol response element-binding protein-1c), HNF4A (hepatic nuclear factor-4), FOXO1 (forkhead box protein 01), PPARG (peroxisome proliferator-activated receptor gamma), FASN (fatty acid synthase), IRS1 (insulin receptor substrate 1), IRS2 (insulin receptor substrate 2), PKLR (pyruvate kinase, liver and red blood cell), ChREBP (carbohydrate response element binding protein), GAPDH (glyceraldehyde phosphate dehydrogenase) and β-actin mRNAs in HepG2 and L-02 cells was examined by RT-PCR. GAPDH and beta-actin were used as controls. Primers for RT-PCR are listed in Table S2 and were synthesized by Takara (Japan). Cells were plated in 24-well plates and total RNA was extracted using Trizol (Life Technologies, USA). 2 µg of RNA was then applied to a reverse transcription reaction using High-Capacity cDNA Reverse Transcription kits (Applied Biosystems, USA) to synthesize cDNA. The PCR protocol to amplify each gene consisted of incubation at 95°C for 5 min, followed by amplification for 40 cycles of three steps at 95°C for 4 min, annealing temperature for 45 sec and 72°C for 45 sec, and then a final extension at 72°C for 10 min. Annealing temperate for the PCR for each gene is listed in Table S2.

### Statistical Analysis

Results are presented as mean ± SD from at least three independent experiments. All luciferase assays were performed in triplicate. Data were analyzed by ANOVA followed by paired Student’s t-tests using the Windows SPSS 13.0 computer software. Differences were considered statistically significant if P<0.05.

## Results

### Conservation of the Structure of Mammalian Glucokinase Genes

**Figure 1:**
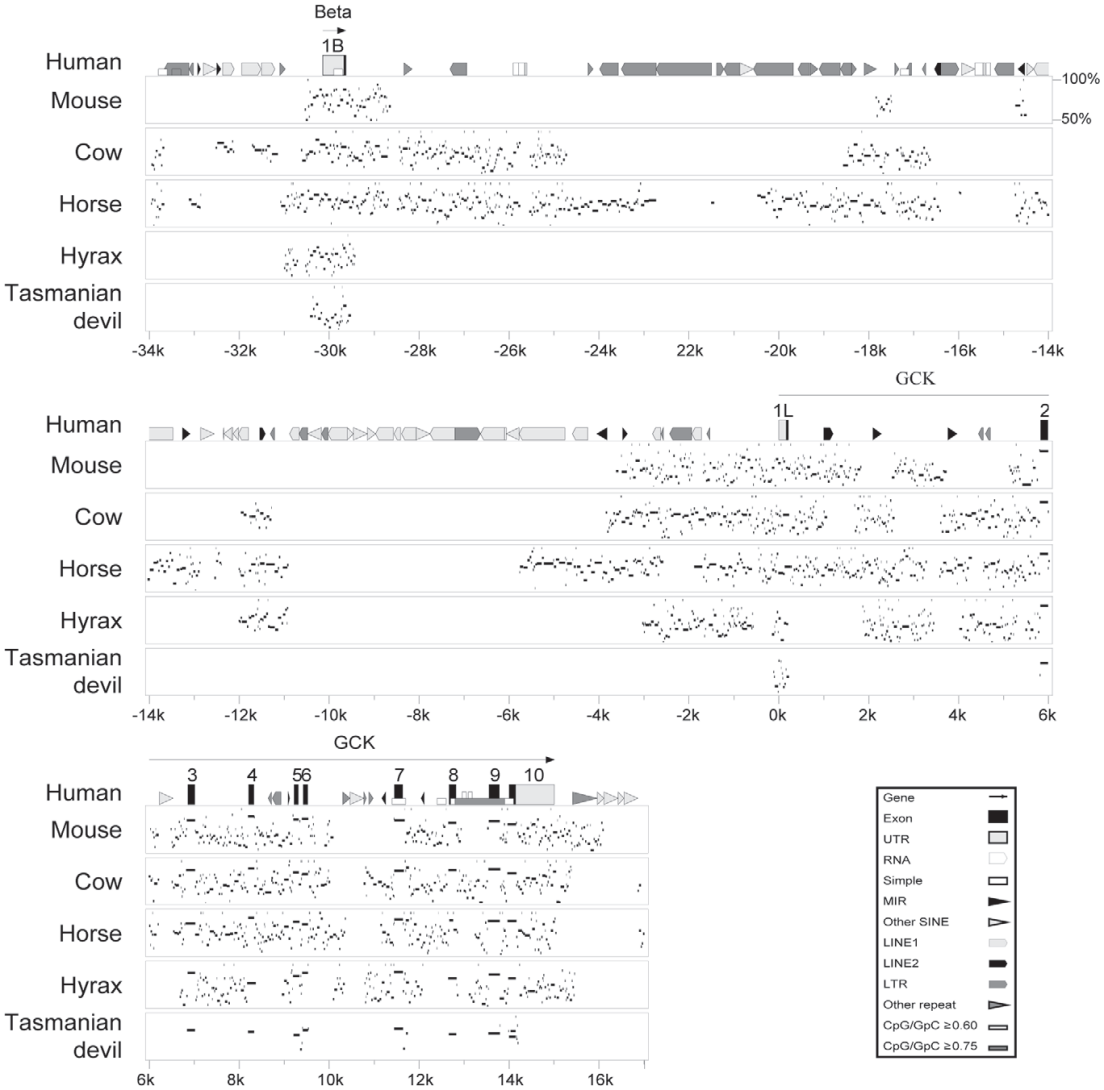
Alignment of glucokinase gene sequences from diverse mammals. A genomic sequence alignment was generated by MultiPipMaker [24], [25]. The sequence is numbered (in kilobases, k) from the 5′ end of the liver-specific transcript, with 5′ flanking sequence numbered backwards. Exons are represented as tall boxes, and are numbered from the 5′ end of the transcripts. The arrow, labeled GCK, represents the liver-specific glucokinase transcript. Beta refers to the 1^st^ exon of the beta-cell-specific GCK transcript, which is spliced to join exon 2. Tissue-specific first exons are labeled as 1B, for the pancreatic beta-cell-specific 1^st^ exon, and 1L, for the liver-cell-specific 1^st^ exon. Filled tall boxes are coding exon sequences, while shaded boxes are untranslated sequences. The percentage sequence identity (if above 50%) of the mouse, cow, horse, hyrax, and Tasmanian devil GCK genomic sequences to the human genomic sequence is shown for each species below the human genomic region schematic. Repetitive DNA elements, and sequence shown high GC content are also identified using the symbols shown in box at the lower right.

Our understanding of the sequences required for the regulation of liver-specific expression of the human glucokinase gene is incomplete [4]. As a first step we compared the structures of glucokinase genes from diverse species. A limited number of glucokinase genes have previously been characterized, where it was found that a pancreatic beta-cell promoter is located 25–30 kb 5′ to a liver-specific promoter [6], [7], [9]. Genomic sequences encoding glucokinase genes were identified and downloaded from all 43 mammalian genomes available in the Ensembl and PreEnsembl genome databases (http://www.ensembl.org) in December 2011 (Table 1). Of the 43 genomes examined, only 19 predict complete glucokinase genes (see Tables 1 and S3) composed of 9 exons that are shared (exons 2–10) by the two transcripts and two alternative initiating 1^st^ exons (beta-cell and liver-specific). Genomes with complete genes spanned the diversity of mammals and included a marsupial and diverse placental mammalian orders (see Fig. 1 and Table 1). Most of the incomplete genes were obtained from genomes that are of draft quality and are composed of short contigs or contigs with gaps, thus missing exons may fall within gaps or beyond the ends of contigs, or may be miss-assembled. An example of a potential miss-assembly is the chimpanzee glucokinase gene, where the beta-cell-specific 1^st^ exon is placed 3′ (instead of 5′) to the coding exons. A potential exception to the conserved structure of the mammalian glucokinase genes is the little brown bat (*Myotis lucifugus*, called Microbat in Ensembl) (Table 1). The genomic sequence encoding the little brown bat glucokinase gene fails to predict a liver-specific 1^st^ exon (Table S3), despite having a contiguous sequence between the beta-cell-specific 1^st^ exon and exon 2, the location where the liver-specific exon is expected to be located. Whether this reflects an incorrect assembly, or the loss of this exon in this bat species requires further study. Intriguingly, bats are one of the few species of mammals that were reported to be deficient in liver glucokinase activity [31].

**Table 1:** Genomic locations of glucokinase genes in diverse mammalian genomes.

Species	Common name	Chromosome/Contig	Coding region^1^	Ensembl ID	Full length^2^
*Homo sapiens*	Human	Chromosome 7	44,184,738–44,228,552	ENSG00000106633	Y
*Pan troglodytes*	Chimpanzee	Chromosome 7	42,808,510–42,850,132	ENSPTRG00000019140	N
*Gorilla gorilla*	Gorilla	Chromosome 7	45,116,842–45,160,953	ENSGGOG00000001501	Y
*Pongo abelii*	Orangutan	Chromosome 7	39,945,143–39,988,782	ENSPPYG00000017596	Y
*Nomascus leucogenys*	Gibbon	GL397320.1	8,862,521–8,906,311	none^3^	Y
*Papio hamadryas*	Baboon	Contig432616_Contig285369	7,819–21,993	ENSP00000223366_1	N
*Macaca mulatta*	Macaque	Chromosome 3	82,145,544–82,190,428	ENSMMUG00000002427	Y
*Callithrix jacchus*	Marmoset	Chromosome 8	11,654,343–11,692,964	ENSCJAG00000008025	Y
*Tarsius syrichta*	Tarsier	GeneScaffold_1825	345–13,054	ENSTSYG00000007022	N
*Otolemur garnettii*	Bushbaby	Scaffold GL873638.1	5,455,542–5,469,662	ENSOGAG00000013533	N
*Microcebus murinus*	Mouse lemur	GeneScaffold_1001	194,109–207,572	ENSMICG00000013503	N
*Mus musculus*	Mouse	Chromosome 11	5,801,858–5,849,602	ENSMUSG00000041798	Y
*Rattus norvegicus*	Rat	Chromosome 14	86,573,358–86,614,433	ENSRNOG00000014447	Y
*Cricetulus griseus*	Chinese hamster	Scaffold JH000506	388,626–426,100	ENSMUST00000109823_1	Y
*Dipodomys ordii*	Kangaroo rat	GeneScaffold_1493	5,956–37,573	ENSDORG00000016392	N
*Cavia porcellus*	Guinea pig	Scaffold_66	2,796,787–2,833,989	ENSCPOG00000005392	N
*Spermophilus tridecemlineatus*	Squirrel	GeneScaffold_577	258,625–272,094	ENSSTOG00000002740	N
*Ochotona princeps*	Pika	GeneScaffold_1053	8,928–17,936	ENSOPRG00000001902	N
*Oryctolagus cuniculus*	Rabbit	Chromosome 10	44,700,788–44,737,207	ENSOCUG00000001018	N
*Bos taurus*	Cow	Chromosome 4	77,821,348–77,856,182	ENSBTAG00000032288	Y
*Ovis aries*	Sheep	Chromosome 4	81,803,493–81,833,770	none	N
*Tursiops truncatus*	Dolphin	GeneScaffold_695	48,308–77,620	ENSTTRG00000006046	Y
*Sus scrofa*	Pig	Chromosome 18	49,522,417–49,565,959	ENSSSCG00000016751	Y
*Vicugna pacos*	Alpaca	Scaffold_90037	954–3,412	none	N
*Equus caballus*	Horse	Chromosome 4	14,992,231–15,041,576	ENSECAG00000022171	Y
*Canis familiaris*	Dog	Chromosome Un	33,595,740–33,634,636	ENSCAFG00000002944	N
*Felis catus*	Cat	GeneScaffold_267	104,622–151,410	ENSFCAG00000014361	N
*Mustela putorius furo*	Ferret	GL896901.1	13,941,619–13,976,233	none	Y
*Ailuropoda melanoleuca*	Panda	GL193092.1	290,421–328,424	ENSAMEG00000017601	Y
*Myotis lucifugus*	Little brown bat(Microbat)	GL430680	118,628–131,052	ENSMLUG00000012016	N^4^
*Pteropus vampyrus*	Flying fox bat(Megabat)	Scaffold_3265	92,412–∼107,000	ENSAMEG00000017601	N
*Tupaia belangeri*	Tree shrew	GeneScaffold_543	134,003–144,092	none	N
*Erinaceus europaeus*	Hedgehog	GeneScaffold_1899	7,736–49,202	ENSEEUG00000001219	Y
*Sorex araneus*	Shrew	Scaffold_232145	2712–7205	ENSSARG00000006856	N
*Loxodonta africana*	Elephant	Scaffold_63	11,963,526–12,101,607	ENSLAFG00000020833	Y
*Procavia capensis*	Hyrax	GeneScaffold_1640	7,777–33,351	ENSPCAG00000010715	Y
*Echinops telfairi*	Lesse hedgehog tenrec	Scaffold_251137	3,251–10,880	ENSETEG00000018598	N
*Dasypus novemcinctus*	Armadillo	GeneScaffold_1550	2,387–31,919	ENSDNOG00000015156	N
*Choloepus hoffmanni*	Sloth	GeneScaffold_1696	4,556–20,427	ENSCHOG00000013302	N
*Monodelphis domestica*	Opossum	Chromosome 1	501,545,523–501,628,991	ENSMODG00000016167	N
*Macropus eugenii*	Wallaby	GeneScaffold_2216	13,891–24,930	ENSMEUG00000001332	N
*Sarcophilus harrisii*	Tasmanian devil	GL834671.1	1,553,525–1,633,424	ENSSHAG00000014686	Y
*Ornithorhynchus anatinus*	Platypus	Contig2861	33,247–48,420	ENSOANG00000006506	N

1 Bases spanning the N-terminus to C-terminus of the encoded protein.

2 Y, indicates that all 11 exons (2 tissue specific and 9 shared) could be predicted from the genomic sequence. N, indicates that at least one exon could not be predicted.

3 None indicates that a gene was not annotated in the genome assembly.

4 A liver specific 1^st^ exon was not found in the genomic sequence, but there is no gap in this sequence.

To further examine the conservation of the structure of glucokinase genes in mammals the sizes of the introns in the glucokinase genes were determined for all 43 species (Table S3). Exon sizes showed minimal size variation (results not shown), as they are largely composed of coding sequence. When present, the intron between the beta-cell-specific 1^st^ exon and exon 2 was always the largest intron, with the intron between the liver-specific 1^st^ exon and exon 2 being the next largest (Table S3). The beta-cell intron was more than 20 kb long in most species, with only four species having an intron length shorter than 10 kb (sloth, 4.6 kb; little brown bat, 6.9 kb; flying fox bat, 8.7 kb; platypus, 9.8 kb – Table S3). For all four of these species the liver-specific exon was not found (Table S3), however for three of these, a gap in the genome assembly exists between the beta-cell exon and exon 2 raising the possibility of having a larger intron (with a liver-specific exon) or miss-assembly. The intron between the liver-specific 1^st^ exon and exon 2 was the second largest (or largest if the beta-cell exon was missing) and ranged in size from 3 to 8.5 kb (Table S3). The remaining glucokinase gene introns (introns 2–9) in all species were much shorter, with most being less than 2 kb and only a few greater than 3 kb (Table S3). A conserved pattern of intron sizes was generally observed with smaller introns (i.e., introns 2, 4, 5, 8, and 9) being smaller in most species, and the larger introns (i.e., beta-cell and liver-specific exon 1, and exons 3, 6, and 7) being larger (Table S3). Introns with the greatest variation in size were the largest introns.

To further examine the genomic sequences encoding glucokinase genes we aligned the genomic sequences using MultiPipMaker [24], [25]. Shown in Figure 1 is an example MultiPipMaker plot aligning the near-complete glucokinase genes from 6 species representing diverse orders of mammals: human (Primates), mouse (Rodentia), cow (Artiodactyla), horse (Perrisodactyla), hyrax (Hyracoidea) and Tasmanian devil (Dasyuromorphia, a marsupial order). MultiPipMaker plots compare genomic sequences to a master sequence (in Fig. 1 the master is the human sequence, with the annotation of the human gene and repetitive DNA content shown on the top line) with the similarity of the compared sequence, between 50–100%, indicated for each species. The sequence from the marsupial Tasmanian devil sequence showed limited similarity to the human genomic sequence (Fig. 1), with similar results found when other genomic sequences were used as the master sequence (see Fig. S2 as an example), with similar sequences confined largely to the coding exons, consistent with most intron and flanking sequence having limited biological function and thus not strongly conserved. When comparisons are limited to between genes from placental mammalian species, many of the intron sequences, especially those of introns 2 through 9, show identities greater than 50%, with exon sequences showing even higher levels of identity (Figs. 1 and S2). Some areas failed to show a similarity above 50%; however, most of these corresponded to gaps in the genomic sequences (and thus have no sequence similarity). In contrast to the 3′ introns, the intronic sequence between the beta-cell specific exon and the liver cell-specific, but not the intron between the liver-specific exon and exon 2, possessed less sequence that had a similarity of 50–100% with the human genomic sequence (Fig. 1). The majority of the low similarity seen at the 5′ end of the gene sequence was not due to gaps in the sequences. Similar results were seen if the mouse, cow, horse or hyrax gene sequence was used as the master sequence (Fig. S2 and results not shown). Similarity of the flanking sequence 5′ to the liver-specific exon was limited to the first 4 kb of the 30 kb sequence between the human liver-specific and beta-cell-specific exons (Fig. 1). The 4 kb of human liver-specific 5′ flanking sequence corresponded to about 6 kb of mouse liver-specific 5′ flanking sequence and about 4 kb of 5′ flanking sequence in the cow, horse, and hyrax (Fig. S2 and data not shown). The more extended mouse 5′ flanking sequence was due to the presence of repetitive DNA elements that had inserted within the 6 kb 5′ flanking sequence that were not orthologous to sequences in the other mammalian gene sequences (Fig. S2).

The presence of repetitive DNA elements explains much of the non-orthology of the sequence between the beta-cell and liver-specific 1^st^ exons. As seen in the MultiPipMaker plots (Figs. 1 and S2) much of the human genomic sequence located between the beta-cell and liver-specific exons is annotated as repetitive DNA. More than 60% of the sequence between these two exons in the human glucokinase gene is derived from repetitive elements, with similar high percentages found for the other mammalian genes examined (Table 2). Among placental mammals, the proportion of the intronic sequence composed of repetitive DNA was much higher between the two tissue-specific exons (37–71%) than between the liver-specific exon and exon 2 (1–23%) or within introns 2–9 (11–21%) (Table 2). Only in the Tasmanian devil, a marsupial, does the sequence between the beta-cell and liver-specific exons have a lower percentage of repetitive DNA (49.7%) than for any other portion of the gene (e.g., 51.1% for the liver-specific exon to exon 2), although this gene has a very high proportion of repetitive DNA across its entire sequence as even introns 2–9 are largely composed (39.8%) of repetitive DNA (Table 2). The high proportion of repetitive DNA in the Tasmanian devil glucokinase gene may in part explain the larger sizes of introns in the gene in this species (Table S3) and for the limited similarity seen in the MultiPipMaker plots (Figs. 1 and S2, and results not shown). A diverse variety and variable number of repetitive DNA elements were found in the genomic sequences between the two tissue-specific 1^st^ exons (Table S4) consistent with most of these elements being inserted into the genes since the radiation of placental mammals. These results suggest that only about 4 kb of sequence 5′ to the liver-specific exon is shared among mammals and only these sequence may contribute to the regulation of gene expression. Most of the sequences between these two tissue-specific 1^st^ exons, especially those more than 4 kb 5′ to the liver-specific mRNA start site is of relatively recent origin from repetitive DNA elements, and thus likely has no role in the regulation of glucokinase gene expression. In contrast to the liver-specific 5′ flanking sequence, most of the sequence downstream of the liver-specific 1^st^ exon is shared among mammals, raising the possibility that these sequences have a role in regulating glucokinase gene expression.

**Table 2:** Repetitive DNA content of introns within mammalian glucokinase genes.

	Beta – Liver^1^		Liver - Intron 2^2^		Introns 2–9^3^	
Species	Size	% Rep	Size	% Rep	Size	% Rep
Human	29,229	60.7	5,609	15.1	12,584	17.0
Mouse	34,421	59.2	3,942	1.3	11,944	18.4
Cow	22,081	37.5	3,949	11.8	11,152	11.4
Horse	36,391	50.3	4,932	22.7	11,417	14.9
Hyrax	8,372	51.8	7,649	22.9	15,606	20.8

1 Sequence between the beta-cell and liver specific 1^st^ exons.

2 Sequence between liver-specific 1^st^ exon and exon 2.

3 Combined sequences of introns 2 through 9.

### Regulatory Activity of the Human Glucokinase Liver-specific 5′ Flanking and Intron 1 Sequences

Since only 4 kb of liver-specific exon 5′ flanking sequence and intron 1 sequences are conserved among mammals, and DNase I hypersensitive sites have been mapped to both of these regions [7], [21], [22] we tested the ability of these sequences to regulate luciferase reporter gene expression in two human liver cell lines. In addition to testing the reporter genes in the well-established HepG2 cell line that has been used in a number of experiments studying the expression of the glucokinase gene promoter [13], [20], [32]–[34], we also tested the constructs in a second human liver cell line, the normal liver cell line L-02 [27]. To examine whether the L-02 cells would be suitable for studying the regulation of glucokinase gene expression we first determined whether L-02 and HepG2 cells express similar profiles of metabolic genes. Expression of GCK, GCKR, components of the insulin signaling pathway (INSR, IRS1, and IRS2), other metabolic enzymes (IPK, PCK1, PYGL, GYS2, and FASN), as well as selected DNA binding proteins involved in the regulation of metabolism (ChREBP, SREBF, NHF4A, PPARG, and FOXO1) were assessed by RT-PCR for cells grown in culture in the presence or absence of insulin or fetal calf serum (Fig. S3). As is typically seen for hepatic cell lines [5], significant expression of the glucokinase gene was not detected in either L-02 or HepG2 cells. Comparing the gene expression profiles of L-02 to HepG2 showed that both cell lines usually express similar levels for most of the genes examined, although slightly higher levels of GCKR and lower levels of SREBF were detected in L-02 (Fig. S3). The increased level of GCRK expression observed by RT-PCR in L-02 cells was confirmed by quantitative real-time RT-PCR (results not shown). RT-PCR experiments showed that there was little change in the expression of the genes due to the presence or absence of insulin or serum for either HepG2 or L-02 cells, except for GCKR in HepG2 (confirmed by quantitative real time RT-PCR, results not shown). It appears that L-02 should be an appropriate cell model system for understanding liver-specific expression of the human glucokinase gene.

**Figure 2:**
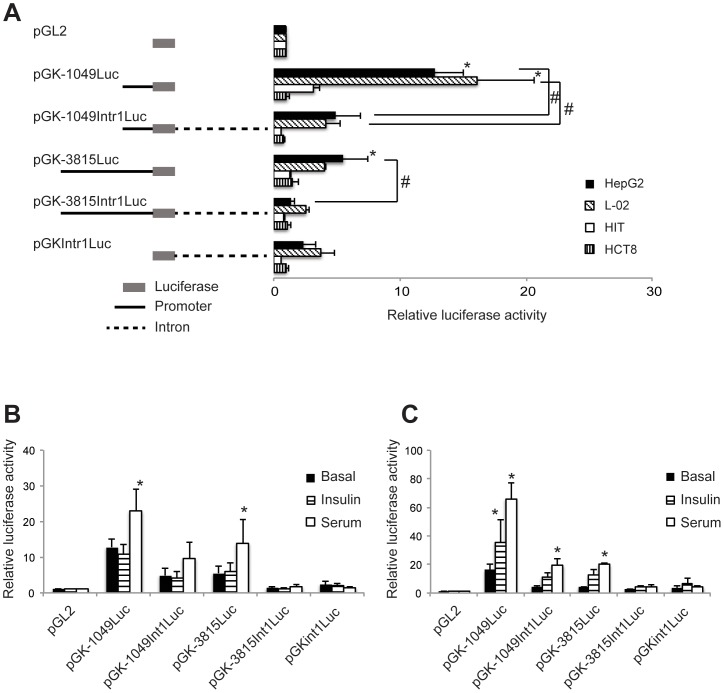
Intron 1 sequences regulate expression of the human glucokinase promoter. A. Schematic illustrations of reporter gene constructs with 1049 or 3815 bases of liver-specific glucokinase 5′ flanking sequence with or without intron 1 are shown on the left. Solid boxes are the luciferase-coding regions. Thin lines represent glucokinase 5′ flanking sequences (1049 or 3815 bases) while the broken lines represent intron 1 sequences. See Fig. S1 for the locations of genomic fragments within the gene. To the right are the luciferase reporter activities detected in two human liver cell lines (L-02 and HepG2), a hamster pancreatic beta-cell line (HIT) and a human intestinal cell line (HCT8). Data are the Mean ± S.D. of three independent experiments. Asterisk (*) indicates reporter gene activity that is significantly greater than that from pGL2 basic. Significant differences in the activity of constructs that differed due to the presence or absence of intronic sequences are indicated by the pound symbol (#), with the comparison shown by the lines (P<0.05). B and C, Responsiveness of the human glucokinase reporter constructs to insulin and fetal calf serum (serum) in HepG2 (B) and L-02 (C) cells. Basal refers to culture without insulin or serum. Constructs from part A were incubated in the presence of absence of 100 nM insulin or 10% fetal calf serum for 20 hours. Data are the Mean ± SD of three independent experiments. Asterisk (*) indicates conditions that have significantly greater activity than the basal level for that construct (P<0.05).

Reporter genes were constructed that contained 1,049 or 3,815 bases of human liver-specific 1^st^ exon 5′ flanking sequence and 135 bases of this exon placed upstream of a luciferase reporter gene (plasmids pGK-1049Luc and pGK-3815Luc, respectively). These two 5′ flanking regions were also placed in vectors that contained 4,658 bases of liver-specific intron 1, plasmids pGK-1049intr1Luc and pGK-3815intr1Luc, with the intronic sequence placed 3′ to the luciferase reporter, thus in a similar position to that found in the endogenous gene. The intron sequences were cloned downstream of the luciferase transcription unit, thus do not need to be spliced from the reporter mRNA to allow translation of the luciferase enzyme. The four reporter gene constructs, along with a construct that contained only the intron sequence cloned downstream of the luciferase reporter (pGKIntr1Luc), were transfected into two human liver cell lines (HepG2 and L-02), a hamster pancreatic beta-cell line (HIT) and a human intestinal cell line (HCT-8) (Fig. 2A). As shown in Fig. 2A significant reporter gene activity was only detected in the two human liver cell lines, indicating that the activity of the reporter constructs was tissue-specific and that the liver-specific promoters do not function in a cell line that represents the beta-cell-specific site of expression (HIT) nor in a cell line derived from a tissue where the glucokinase gene is not normally expressed (HCT-8). Strongest expression of the reporter gene was observed for the construct that contained only the 1049 bases of glucokinase 5′ flanking sequence, however the construct with 3,815 bases of 5′ flanking sequence also supported significant levels of reporter expression in HepG2 cells (Fig. 2A). The lower levels of expression with 3,815 base of 5′ flanking sequence may suggest that negative regulatory elements reside in the sequence between bases −1049 and −3815. Intriguingly, addition of intron 1 sequence to either 1049 or 3815 bases of 5′ flanking sequence (pGK-1049intr1Luc and pGK-3815intr1Luc, respectively) resulted in significantly reduced levels of reporter gene expression in HepG2 cells (Fig. 2A). For the L-02 cells, a similar significant repression was observed with the reporter construct with 1049 bases of 5′ flanking sequence, but since the 3815 base construct by itself did not generate a significant increase over background the observed reduction for the plasmid with the intron sequences was not significant (Fig. 2A). As the intron sequences are not part of the reporter gene transcript, these results suggest that the sequences within intron 1 act to repress expression of the liver-specific glucokinase promoter.

### Regulation of Liver-specific Glucokinase Promoters by Insulin and Fetal Calf Serum

Expression of the glucokinase gene in the liver is absolutely dependent upon the presence of insulin [4], [9]. We tested the activity of our 5′ flanking region and intron containing reporter gene constructs under basal conditions and after exposure to 100 nM insulin or 10% fetal calf serum for 20 hours (Figs. 2B and 2C). As has been previously reported [9], glucokinase reporter gene constructs in HepG2 cells were unresponsive insulin, but did show a statistically significant increase (P<0.05) in the presence of fetal calf serum for the plasmids pGK-3815Luc and pGK-1049Luc (Fig. 2B). A small increase with fetal calf serum was seen in the pGK-1049Intr1Luc intron-containing construct, but it did not reach statistical significance and the intron containing construct with the longest 5′ flanking region, pGK-3815Intr1Luc had minimal activity under all treatments (Fig. 2B). In contrast to HepG2, a significant (P<0.05) increase in reporter gene activity was observed with the pGK-1049Luc promoter construct in response to insulin (Fig. 2C). Small increases were also observed with the pGK-3815Luc and pGK-1049Intr1Luc constructs, but they were not statistically significant. When the constructs were treated with fetal calf serum in the L-02 cells, significant increases in reporter gene activity were seen with the pGK-1049Luc, pGK-3815Luc, and pGK-1049Intr1Luc constructs (Fig. 2C). Since the introduced human liver-specific glucokinase promoter could be induced by insulin, these results suggest that the L-02 cell line might be a better model for understanding the mechanisms by which insulin regulates liver-specific glucokinase promoter function.

### Regulatory Elements in the 5′ Flanking Sequence of the Human Liver-specific Glucokinase Promoter

**Figure 3:**
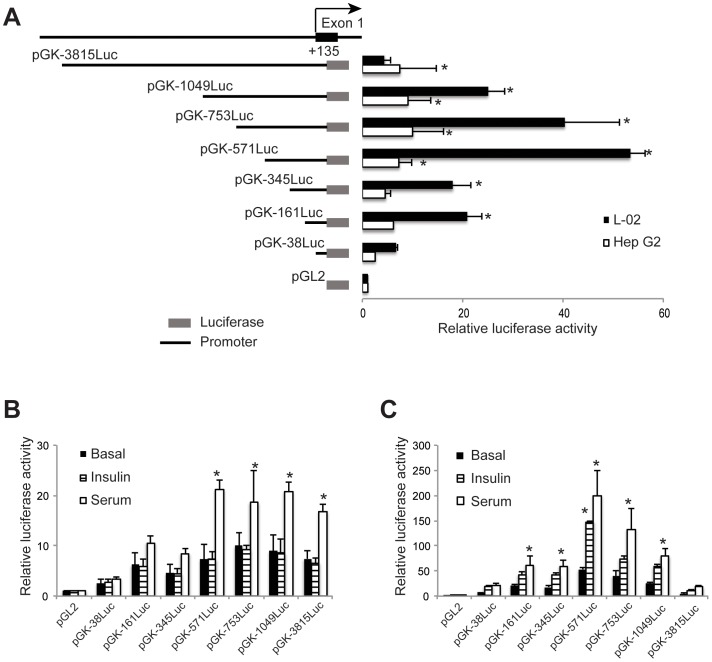
Transcriptional activity of deletions of the human glucokinase 5′ flanking sequence. A. Schematic illustrations of the human glucokinase promoter deletion constructs used to test promoter activity are shown on the left. See Fig. S1 for the locations of genomic fragments within the gene. Reporter gene activity in two human cell lines, HepG2 and L-02 is shown on the right. Asterisk (*) indicates reporter gene activity that is significantly greater than that from pGL2 basic. Data are the Mean ± SD of three independent experiments. B and C. Responsiveness of the human glucokinase promoter constructs to insulin and fetal calf serum (serum) in HepG2 (B) and L-02 (C) cells. Basal refers to culture without insulin or serum. Constructs from part A were incubated in the presence of absence of 100 nM insulin or 10% fetal calf serum for 20 hours. Data are the Mean ± SD of three independent experiments. Asterisk (*) indicates conditions that have significantly greater activity than the basal level for that construct (P<0.05).

To better define the sequences in the 5′ flanking sequence of the human liver-specific glucokinase promoter necessary for expression and for regulation by insulin and fetal calf serum a series of reporter constructs were generated that have differing lengths of 5′ flanking sequence. The activities of the promoter deletion constructs were tested in human liver HepG2 and L-02 cell lines (Fig. 3). Since 1,049 bases of 5′ flanking sequence generated more reporter gene activity than plasmids with 3,815 bases (Fig. 2A), implying the presence of negative regulatory sequences upstream of base −1049, we focused on deletions starting from base −1049. Greater activity was observed in L-02 cells than in HepG2 cells, except for the longest construct pGK-3815Luc (Fig. 3A). Reporter gene constructs with as little as 161 bases and as much as 1049 bases of 5′ flanking sequence supported significant reporter gene activity in L-02, but only constructs with −571 bases or more, including the construct with 3815 bases, supported significant reporter activity in HepG2 cells (Fig. 2A). These results suggest that there are two areas in the liver-specific glucokinase 5′ flanking sequence that provide positive activity: 1, the sequence between bases −38 and −161, which supports activity in L-02 cells; and 2, the sequence between bases −345 and −571, which is required by HepG2 cells. The region between bases −345 and −571 likely also contributes to the greater activity of the pGK-571Luc construct, compared to the pGK-345Luc construct, in L-02 cells (Fig. 3A). Evidence for several negatively acting regions located upstream of base −571 was also found (Fig. 3A). Maximum reporter gene activity was observed with the pGK-571Luc construct in L-02 cells with lower activity observed with the pGK-753Luc, pGK-1049Luc, and pGK-3815Luc constructs in L-02, with the pGK-3815Luc construct having a level of reporter activity that was not significantly greater than that of a vector without a promoter (pGL2) (Fig. 3A). These results suggests that negative acting sequences exist in the human glucokinase liver-specific 5′ flanking sequence.

We also tested the ability of the shorter promoters to be regulated by insulin and fetal calf serum (Figs. 3B and 3C). In HepG2 cells, addition of insulin did not change the level of reporter gene expression of any of the glucokinase promoter constructs (Fig. 3B). Treatment with fetal calf serum, on the other hand, resulted in a significant enhancement of reporter gene expression in promoter constructs that had at least 571 bases of 5′ flanking sequence (Fig. 3B). These results suggest that sequences responsive to a factor in fetal calf serum are located between bases −345 and −571. In contrast to HepG2, insulin increased the expression of glucokinase promoters in the L-02 cell line (Fig. 3C). A significant increase in reporter gene activity was observed in the presence of insulin with the pGK-571Luc construct, indicating that an insulin responsive element is located between bases −345 and −571 of the human glucokinase liver-specific promoter. Increases in reporter activity were also seen with the −753 and −1049 base reporter constructs, but the increase did not reach statistical significance in this experiment (Fig. 3C). Similarly, increases in reporter activity were seen with glucokinase promoter constructs when L-02 cells were treated with fetal calf serum (Fig. 3C). A significant increase was seen with as little as 161 bases of 5′ flanking sequence, however the maximum increase was seen with 571 bases of sequence and smaller increases with longer promoter constructs (Fig. 3C). These results suggest that an element response to a factor found in serum resides between bases −38 and −161 in the human liver-specific glucokinase promoter, and that potentially a second site, which may respond to a different factor present in serum, is located between bases −345 and −571. In addition, these results suggest that sequences upstream of base −571 have a negative effect on both the insulin and serum responsiveness of the promoter constructs in the L-02 cell line.

### Regulatory Elements in the Liver-specific Intron 1 of the Human Glucokinase Gene

**Figure 4:**
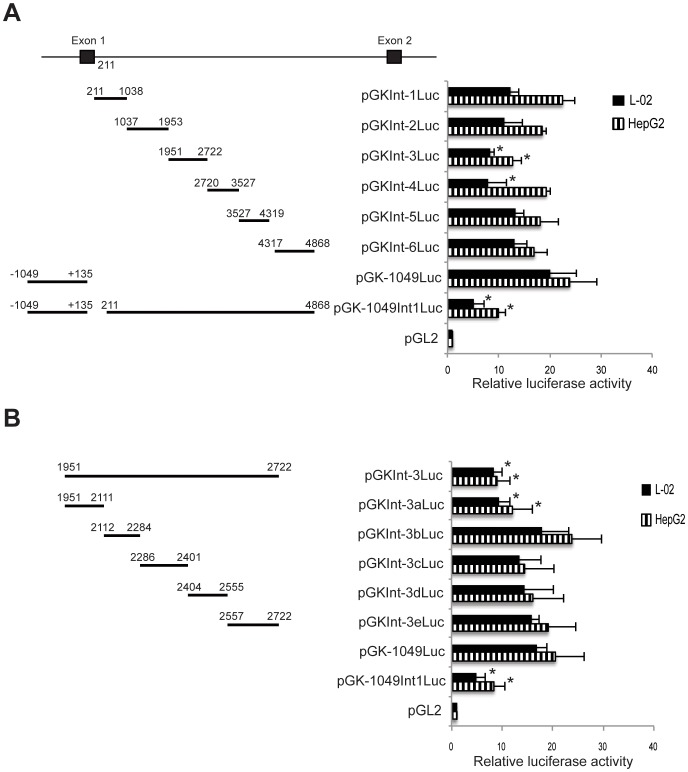
Analysis of the transcriptional activity of fragments of intron 1 of the human GCK gene. A. Activity of large fragments spanning most of intron 1. B Activates of subfragments of intronic fragment Int-3 (I-3). Schematics on the left indicate the relative positions of fragments of intron 1 inserted downstream of a luciferase reporter gene in the pGK-1049Luc reporter plasmid. See Fig. S1 for the locations of genomic fragments within the gene. Reporter gene activities of the constructs in human L-02 and HepG2 cells are shown on the right. Data are the Mean ± SD of three independent experiments. Asterisk (*) indicates constructs that have significantly different activity than the −1049 construct (P<0.05).

When sequences from intron 1 are placed downstream of the luciferase coding sequence lower reporter gene activity was observed for both the 1049 and 3815 base liver-specific glucokinase promoter constructs (Fig. 2A). To determine which portion of the intron confers this negative activity we subcloned 6 fragments of the intron (I-1 to I-6, see Fig. S1) and placed them downstream of the luciferase reporter gene in pGK-1049Luc. The reporter gene activity of these constructs are shown in Fig. 4A. In HepG2, 5 of the 6 fragments show reporter activity levels similar to that of pGK-1049Luc, with only one fragment, fragment I-3, showing significantly lower reporter gene activity, and yielding reporter activity levels that are similar to those of the pGK-1049intr1Luc construct (Fig. 4A). Similarly, in L-02, fragment I-3, yielded significantly lower reporter gene activity, but in contrast to HepG2, fragment I-4 also had significantly lower reporter gene activity (Fig. 4A). These results suggest that sequences in intron 1 between bases 1951 and 2722 of the human glucokinase gene function to repress gene expression, with the possibility that a second sequence between bases 2720 and 3527 (fragment I-4) also have some activity, at least in some types of liver cells (e.g., L-02, Fig. 4A).

To better define the sequences that confer the negative regulatory activity in both liver cell lines, we subcloned smaller sections of intron fragment I-3 (fragments a–e, Fig. S1) and placed them downstream of the luciferase reporter gene in pGK-1049Luc (Fig. 4B). In both L-02 and HepG2 only one of the DNA subfragments, fragment 3a containing bases between 1951 and 2111, yields significantly lower reporter gene activity than the pGK-1049Luc vector, and this fragment generates a level of reporter activity that is similar to that of constructs containing all of the intron 1 sequences (pGK-1049Int1Luc) or fragment I-3 (pGKInt-3Luc) (Fig. 4B). These results suggest that sequences between bases 1951 and 2111, relative to the liver-specific mRNA start site, negatively regulate liver-specific glucokinase gene expression.

**Table 3:** Potential transcription factor binding sites near the human glucokinase liver-specific 1^st^ exon.

Position^1^	Transcription factor^2^
Promoter (−345 to −571)	
−561 to −537	HNF4, hepatic nuclear factor-4
−520 to −496	HNF4, hepatic nuclear factor-4
−476 to −460	HFH, forkhead domain factor
−411 to −395	HNF-6, onecut, hepatic nuclear factor-6
−361 to −345	ChREBP, carbohydrate repose element binding protein
−360 to −334	CTCF, CCCTC-binding factor
Promoter (−38 to −161)	
−163 to −125	TFIID, transcription factor II D
−156 to −136	YY, yin-yang 1
−153 to −137	HFH, forkhead domain factor
−101 to −79	PPAR_RXR, peroxisome proliferator-activated receptor retinoid X receptor
−68 to −44	PPAR_RXR, peroxisome proliferator-activated receptor retinoid X receptor
Intron (1951 to 2111)	
1969 to 1995	CTCF, CCCTC-binding factor
1981 to 1997	ChREBP, carbohydrate response factor binding protein
2012 to 2022	SMAD, Sma and Mad (Mothers against decapentaplegic)
2016 to 2034	STAT, Signal transducer and activator of transcription
2068 to 2090	PPARG, peroxisome proliferator-activated receptor gamma

1 Location of the binding site relative to liver-specific mRNA start site.

2 Name of protein that potentially binds to the human glucokinase genomic sequence.

## Discussion

Human and rodent glucokinase genes possess two promoters that are utilized in a tissue-specific manner [6]–[8] with the 5′ most promoter used by pancreatic beta-cells, and a few cells in the gut and central nervous system, and a 3′ promoter that is used exclusively by liver cells [4], [9]. As little as 294 bases of beta-cell-specific promoter 5′ flanking sequence directs tissue-specific expression [10], [11], however, the minimal sequences required for liver-specific expression have not been identified [4]. The only transgenic construct that achieved liver-specific glucokinase expression contains 83 kb of the human glucokinase locus [12] and transgenic mice with only 7.5 kb of human glucokinase liver-specific 5′ flanking sequence did not express the reporter gene in the liver. These observations suggest that sequences either further 5′ than −7.5 kb or downstream of the liver-specific mRNA start site are required for liver-specific expression [7].

To address the question concerning what sequence might be necessary for liver-specific expression we choose to better understand the structure of glucokinase genes in diverse mammals. Understanding the conservation of genomic sequences should allow us to determine whether sequences upstream or downstream of the liver-specific 1^st^ exon contribute to liver-specific gene expression. We identified 19 complete glucokinase gene sequences from the 43 mammalian species with available genome sequences (from www.ensembl.org, Table 1). From these genomic sequences we found that all, except possibly the little brown bat, had identifiable beta-cell and liver-specific 1^st^ exons (Tables 1 and S3) suggesting that tissue-specific promoters exist in most if not all mammals. The distance between the beta-cell and liver-specific 1^st^ exons in the 20 genes is fairly large, and similar to the previously reported sizes, of about 30 kb, for the human and rodent genes [6]–[8] (Table S3). Intriguingly, when the genomic sequences were aligned (see Figs. 1 and S2) little sequence similarity is seen in the genomic sequences between the two tissue-specific 1^st^ exons, in contrast to the remainder of the gene sequences where considerable similarity is observed between the genomic sequences encoding glucokinase from diverse placental mammals. A more careful examination of the genomic sequences demonstrated that most of the sequence between the two tissue-specific 1^st^ exons is composed of repetitive DNA elements (Tables S3 and S4), and that these elements were likely inserted into the genomic sequences independently in the divergent orders of mammals. The discovery that most of the sequence between the tissue-specific exons is recently inserted repetitive DNA suggests that any regulatory sequence in this region should be in the non-repetitive regions. Examination of the genomic sequence alignments indicates that only about 4 kb of the human genomic sequence 5′ to the liver-specific exon is non-repetitive (Fig. 1). Since the 4 kb region was included in the previously tested transgenic mouse (which had 7.5 kb of liver-specific 5′ flanking region) that did not express the reporter gene in the liver [7], suggests that the sequences necessary for liver-specific expression must be located 3′ to the mRNA start site of the liver-specific glucokinase transcript.

Alignments of the glucokinase genomic sequences demonstrates that sequences downstream of the liver-specific 1^st^ exon are similar in diverse mammals, except in the comparisons with the Tasmanian devil, a marsupial species that has had the most time to allow sequence divergence (Figs. 1 and S2). DNase I hypertensive sites have been mapped to locations both 5′ and 3′ to the liver-specific 1^st^ exon [7], [21], [22]. DNase I hypertensive sites are often associated with sequences that regulate gene expression, however, the 3′ site has not been tested in previous analyses of liver-specific glucokinase gene expression [35], [36]. The conservation of the intronic sequences downstream of the liver-specific 1^st^ exon and the presence of a DNase I hypersensitive site within this region suggests these sequences may play a role in the liver-specific expression of the glucokinase gene.

To test whether intron sequences downstream of the liver-specific 1^st^ exons have a role in the expression of the glucokinase gene we generated reporter gene constructs containing liver-specific 5′ flanking sequences with or without intron sequences (see Fig. S1). Our in vitro transfection experiments show that reporter gene plasmids that contain either 3815 or 1049 bases of liver-specific 5′ flanking sequence generate lower levels of reporter gene expression if intron 1 sequences were included in the reporter constructs (Fig. 2). These results suggest that sequences in the intron downstream of the liver-specific 1^st^ exon contribute to the downregulation of expression of the glucokinase gene. Hormones regulate the expression of the glucokinase gene [4], [9], thus we tested the ability of insulin and fetal calf serum to modify expression of our reporter gene constructs. In previous studies, insulin failed to induce expression of the endogenous glucokinase gene, or introduced glucokinase promoter reporter genes in HepG2 cells [4], [9], and with these cells we observed similar results (Fig. 2B). Fetal calf serum, however increased expression of our reporter gene constructs containing 5′ flanking, but not intron, sequences suggesting that a factor(s) present in serum induced expression (Fig. 2B). Similar results were seen with fetal calf serum using these constructs in L-02 cells (Fig. 2C). Fetal calf serum is a complex mixture and includes many factors such as growth factors and hormones that could induce gene expression. For example, estrogen has been found to regulate the rat glucokinase gene expression via sequences near the mRNA star site [37]. In contrast to HepG2, induction of reporter gene expression by insulin was observed in the normal liver cell line L-02, although this was only seen with some of the 5′ flanking sequence-containing constructs (Figs. 2C and 3C). Constructs that contained additional 5′ flanking sequence (e.g., pGK-3815Luc) or intron sequences (e.g., pGK-1049Intr1Luc) were not induced by insulin suggesting that sequences further 5′ or within intron 1 may modulate the activity of insulin. This report, however, is the first to demonstrate that an introduced glucokinase promoter construct can be regulated by insulin in a liver cell line, the L-02 cell line [27], thus the use of this cell line should increase our ability to understand the factors responsible for insulin regulated liver-specific glucokinase gene expression.

To better characterize the sequences necessary for both expression of the reporter constructs in liver cells and it’s down regulation, deletions of the 5′ flanking and intron regions were made. Differing results were observed with the HepG2 and L-02 liver cell lines for the deletions of 5′ flanking sequence (Fig. 3A). HepG2 cells require at least 571 bases of 5′ flanking sequence to generate significant reporter gene expression, while L-02 cells only needed 161 bases (Fig. 3A). As glucokinase reporter gene construct expression in L-02 cells is responsive to insulin (Figs. 2 and 3), and hepatic glucokinase gene expression is absolutely dependent on the presence of insulin [4], [9], we suggest that the results derived from the L-02 cell line may be more representative of endogenous gene expression in the liver. The minimal sequence necessary for reporter gene expression in L-02 cells is similar to length (180 bases) of rat glucokinase liver-specific promoter required for expression in rat hepatocytes [36]. Insulin induced expression of reporter gene constructs only in the L-02 cells, however only the construct with 571 bases of 5′ flanking sequence were significantly stimulated by insulin (Fig. 3C). Reporter constructs with 753 and 1049 bases of 5′ flanking sequence, while having increased reporter gene expression in L-02 cells when exposed to insulin, did not have increases that were statistically significant (Fig. 3C). These observations may suggest that sequences upstream of −571 may modulate the function of the insulin responsive element, and thus multiple sequences may act to negatively regulate glucokinase gene expression. In addition to the negative regulatory sequences, these results suggest that sequences at two locations are involved in positively enhancing the expression of the glucokinase promoter, one between bases −38 and −161 necessary for basal expression and one between −345 and −571 that can be regulated by insulin. Searches for potential transcription factor-binding sites between bases −345 and −571 identified a number of potential factors (Table 3) and these regions show conservation in sequence, and potential transcription factor binding sites, between diverse mammalian species (Fig. 1). Among the candidate sites are several that have been identified to have roles in the liver-specific expression of glucokinase including hepatic nuclear factors-4 (HNF-4) and -6 (HNF-6) [20], [38], [39]. A site that potentially interacts with forkhead factors, such as FKHR and Foxo1 [20], [40] that also regulate glucokinase was also found (Table 3). In the proximal region, between bases −38 and −161, in addition to a forkhead factor-binding site, potential binding sites for PPAR and RXR, factors that are important in hepatic lipid metabolism [41], were found (Table 3). In addition, the proximal sequence contains a binding site for the core transcription factor TFIID [42] (Table 3).

A 160-base-long sequence about 2000 bases downstream of the liver-specific transcription start site was found to repress expression driven by the 5′ flanking sequence of the glucokinase liver-specific 1^st^ exon (Fig. 4). A conserved sequence was also found in the genomic sequences of diverse mammals, except the mouse (Fig. 1). Intron 1 sequences repressed activity of both the 1049 and 3815 base long glucokinase liver-specific 5′ flanking sequence, although it had its greatest effect on the most strongly expressed flanking sequence (Fig. 2). The large and short intron sequences had repressor activity in both HepG2 and L-02 cells, and a single sequence may explain the complete activity (Figs. 2 and 4). Within the 160-base-long minimal element several potential transcription factor-binding sites were identified (Table 3), with many of these sites showing conservation between diverse mammals. Among these are sites for ChREBP and PPARG, factors important in liver metabolism [16], [43], sites for signaling proteins SMAD and STAT [44] and the insulator protein CTCF [45]. These sites give the potential that this region could integrate signals to regulate glucokinase gene expression.

## Supporting Information

Figure S1 **Genomic fragments used to test liver-specific glucokinase promoters.** Schematic illustrations of genomic fragments amplified to test glucokinase promoters. The top line illustrates the genomic organization of the 5′ end of human liver-specific glucokinase gene region. Exons are shown as boxes, with coding region as a filled box and untranslated region as an open box. Intron and flanking sequence are indicates as a thin line. The relative sizes and locations of amplified fragments are shown (see Table S1 for primers used for their construction). 5′ flanking fragments are labeled with the 5′ base. Intron fragments are labeled I, with subfragments of I-3 labeled 1 a–e.
(TIF)

Figure S2 **Alignment of glucokinase gene sequences from diverse mammals, using mouse as the master sequence.** A genomic sequence alignment was generated by MultiPipMaker (24,25). The sequence is numbered (in kilobases, k) from the 5′ end of the liver-specific transcript, with 5′ flanking sequence numbered backwards. Exons are represented as tall boxes, and are numbered from the 5′ end of the transcripts. The arrow, labeled GCK, represents the liver-specific glucokinase transcript. Beta refers to the 1^st^ exon of the beta-cell-specific GCK transcript, which is spliced to join exon 2. Tissue-specific first exons are labeled as 1B, for the pancreatic beta-cell-specific exon, and 1L, for the liver-cell-specific exon. Filled tall boxes are coding exon sequences, while shaded boxes are untranslated sequences. The percentage sequence identity (if above 50%) of the human, cow, horse, hyrax, and Tasmanian devil GCK genomic sequences to the mouse genomic sequence are shown for each species below the mouse genomic region schematic. Repetitive DNA elements, and sequence shown high GC content are also identified using the symbols shown in box at the lower right.
(TIF)

Figure S3 **Characterization of the human L-02 normal liver cell line.** Expression of genes involved in glucose metabolism in HepG2 and L-02 cell lines was assed by RT-PCR (primers listed in Table S2). Cells were tested under basal conditions or after stimulation by 100 mM insulin or 10% fetal calf serum for 20 hours. Beta-actin and GAPDH were used as controls.
(TIF)

Table S1 **Primers used to generate human glucokinase reporter gene constructs.**
(DOCX)

Table S2 **Primers used for the RT-PCR analysis of gene expression in HepG2 and L-02 cell lines.**
(DOCX)

Table S3 **Sizes of introns (in bp) in mammalian glucokinase genes.**
(DOCX)

Table S4 **Number and amount of repetitive DNA elements located between the beta-cell and liver-specific exons of mammalian glucokinase gene.**
(DOCX)
