# Supplementary figures and images for: Expression of the Human Glucokinase Gene: Important Roles of the 5′ Flanking and Intron 1 Sequences
Source: PLoS One. 2012 Sep 20;7(9):e45824. doi: 10.1371/journal.pone.0045824 (PMC3447760; doi:10.1371/journal.pone.0045824)

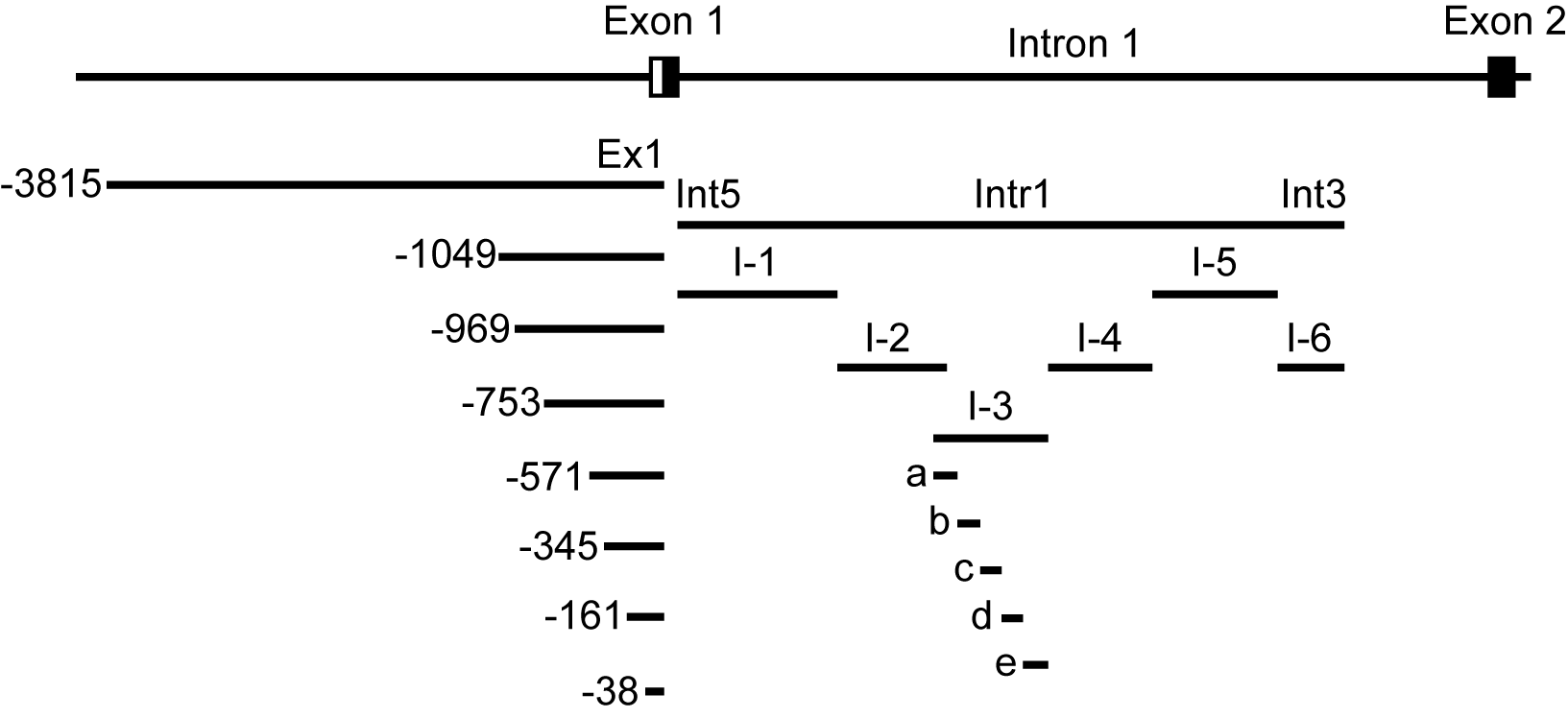

Supplement: Figure S1 — Genomic fragments used to test liver-specific glucokinase promoters. Schematic illustrations of genomic fragments amplified to test glucokinase promoters. The top line illustrates the genomic organization of the 5′ end of human liver-specific glucokinase gene region. Exons are shown as boxes, with coding region as a filled box and untranslated region as an open box. Intron and flanking sequence are indicates as a thin line. The relative sizes and locations of amplified fragments are shown (see Table S1 for primers used for their construction). 5′ flanking fragments are labeled with the 5′ base. Intron fragments are labeled I, with subfragments of I-3 labeled 1 a–e. (TIF) [file pone.0045824.s001.tif]

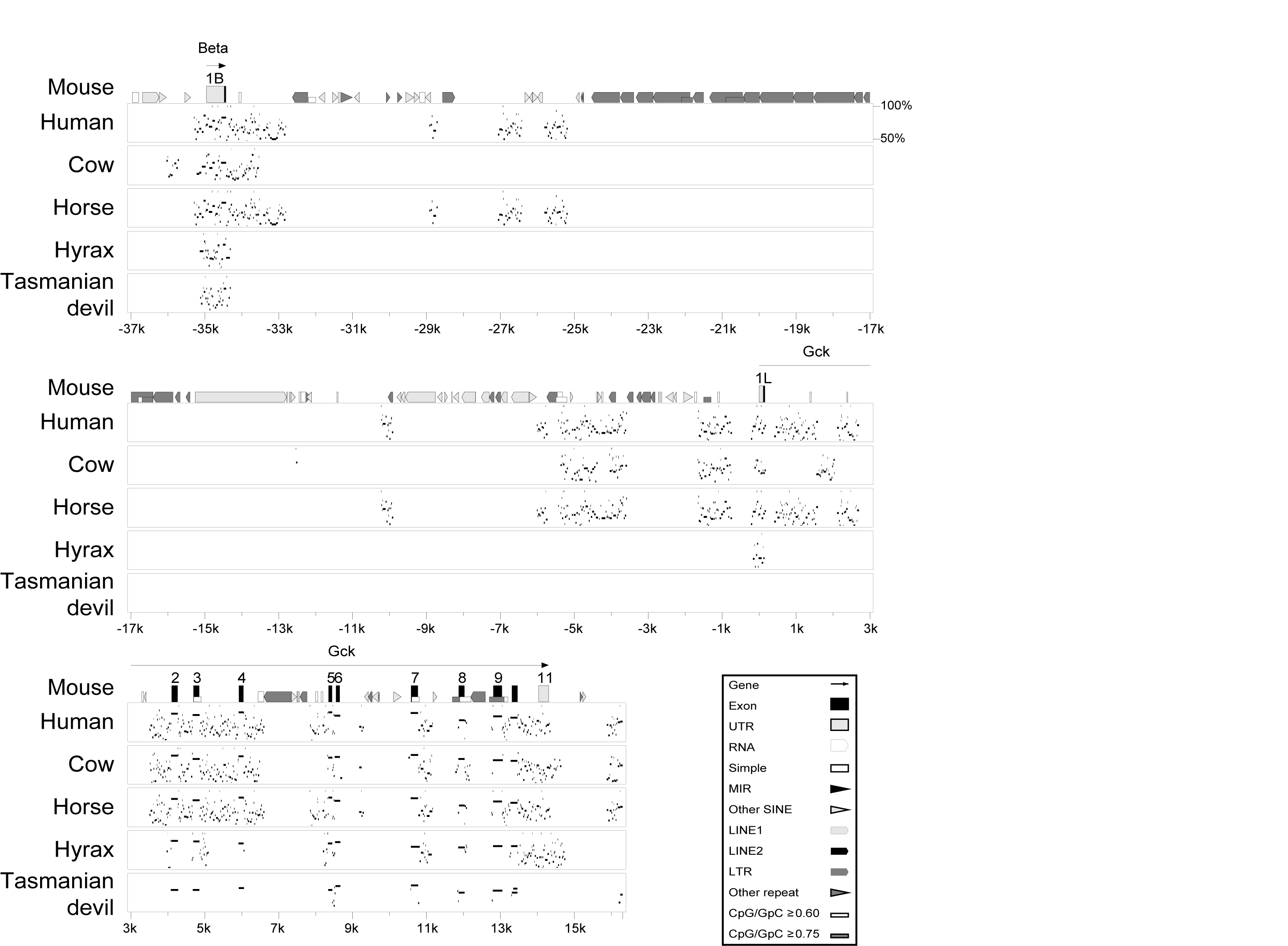

Supplement: Figure S2 — Alignment of glucokinase gene sequences from diverse mammals, using mouse as the master sequence. A genomic sequence alignment was generated by MultiPipMaker (24,25). The sequence is numbered (in kilobases, k) from the 5′ end of the liver-specific transcript, with 5′ flanking sequence numbered backwards. Exons are represented as tall boxes, and are numbered from the 5′ end of the transcripts. The arrow, labeled GCK, represents the liver-specific glucokinase transcript. Beta refers to the 1st exon of the beta-cell-specific GCK transcript, which is spliced to join exon 2. Tissue-specific first exons are labeled as 1B, for the pancreatic beta-cell-specific exon, and 1L, for the liver-cell-specific exon. Filled tall boxes are coding exon sequences, while shaded boxes are untranslated sequences. The percentage sequence identity (if above 50%) of the human, cow, horse, hyrax, and Tasmanian devil GCK genomic sequences to the mouse genomic sequence are shown for each species below the mouse genomic region schematic. Repetitive DNA elements, and sequence shown high GC content are also identified using the symbols shown in box at the lower right. (TIF) [file pone.0045824.s002.tif]

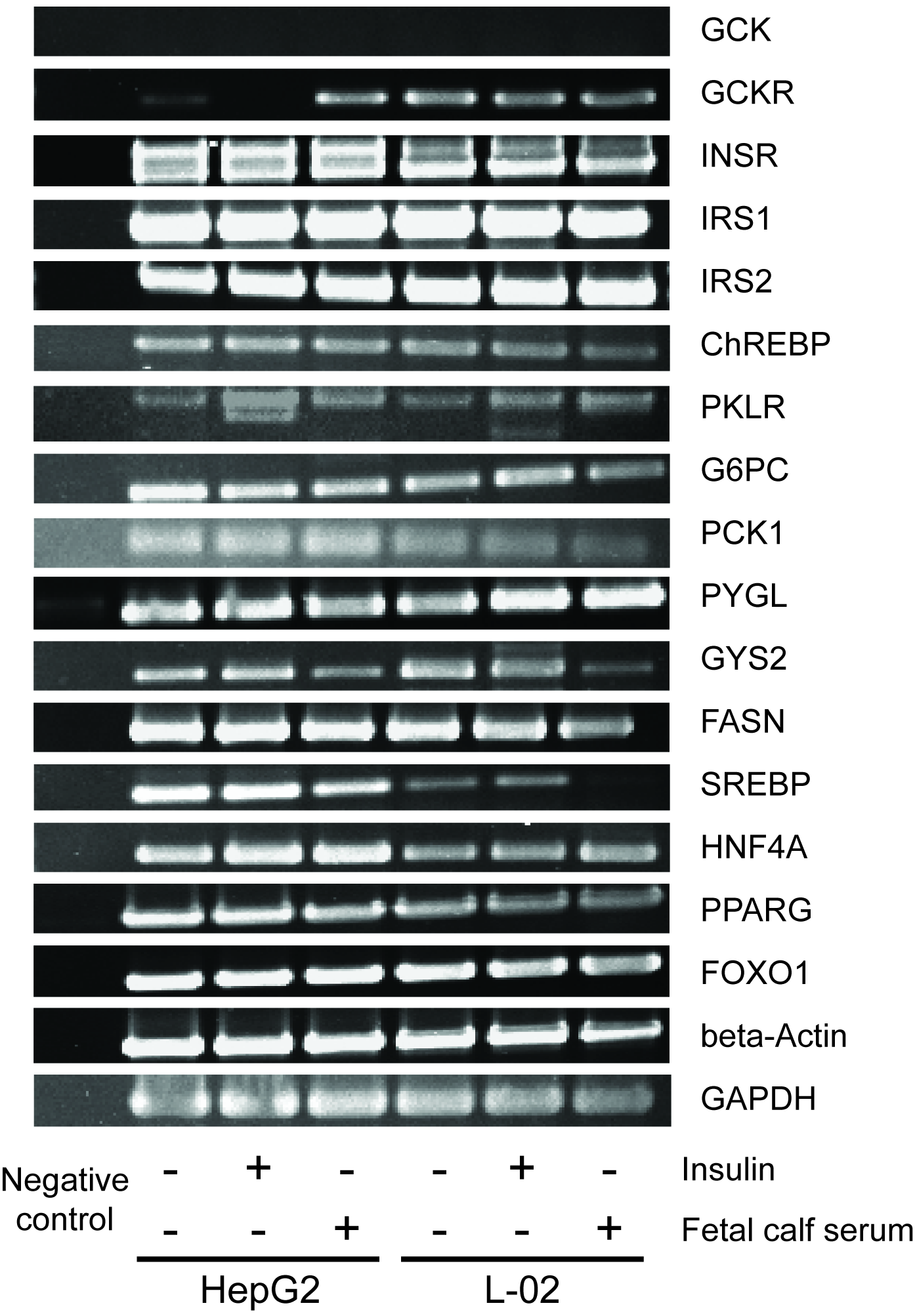

Supplement: Figure S3 — Characterization of the human L-02 normal liver cell line. Expression of genes involved in glucose metabolism in HepG2 and L-02 cell lines was assed by RT-PCR (primers listed in Table S2). Cells were tested under basal conditions or after stimulation by 100 mM insulin or 10% fetal calf serum for 20 hours. Beta-actin and GAPDH were used as controls. (TIF) [file pone.0045824.s003.tif]
