# Supplementary material for: Expression of the Human Glucokinase Gene: Important Roles of the 5′ Flanking and Intron 1 Sequences
Source: PLoS One. 2012 Sep 20;7(9):e45824. doi: 10.1371/journal.pone.0045824 (PMC3447760; doi:10.1371/journal.pone.0045824)
Supplement: Table S1 — Primers used to generate human glucokinase reporter gene constructs. (DOCX) [file pone.0045824.s004.docx]

**Table S1. Primers used to generate human glucokinase reporter gene constructs.**

| **Name** | **Location^1^** | **Strand^2^** | **Sequence^3^** | **RE Site^4^** |
| --- | --- | --- | --- | --- |
|  | Promoter |  |  |  |
| -3815f | -3,815 | + | ggcgagctcACAGAGTCTTGGATGATTCTC | SacI |
| -1049f | -1,049 | + | ggcgagctcCCACTTGCCTCAGCTTCAGGC | SacI |
| -753f | -753 | + | ggcgagctcAAGGGCTTTCTTGGGAGTGAT | SacI |
| -571f | -571 | + | ggcgagctcAGAGCCTGGAAGTCATGGTCT | SacI |
| -345f | -345 | + | ggcgagctcCCTCCAAGAGCAAGTCCAGAC | SacI |
| -161f | -161 | + | ggcgagctcATCCCTACCCCATGTTCACAG | SacI |
| -38f | -38 | + | ggcgagctcGGCAGAGTATTTGAGC | SacI |
| Ex1 | 135 | - | ggcaagcttTTTGGGAGGCAGAGATGCTCC | HindIII |
|  | Intron |  |  |  |
| Int5 | 211 | + | ggcgtcgacGTAAGGGTCACACCAAAGTTAG | SalI |
| I-1f | 211 | + | gcgtcgacGTAAGGGTCACACCAAAG | SalI |
| I-1r | 1,308 | - | cgggatccTTGGTGAAAGCGATAAG | BamHI |
| I-2f | 1,307 | + | gcgtcgacAAGCCTGCGCCCA | SalI |
| I-2r | 1,950 | - | cgggatccTCCCAAGCAGTGTAGGAA | BamHI |
| I-3f | 1,951 | + | ggcgtcgacAAGCCCTGTAAGTTTGC | SalI |
| I-3ar | 2,111 | - | gggatccTTGAACCCAGGCACTGG | BamHI |
| I-3bf | 2,112 | + | cgtcgacACTCCTCTGTGGCCTATGGT | SalI |
| I-3br | 2,284 | - | cgggatccCTGATGCACACTTCCTG | BamHI |
| I-3cf | 2,285 | + | gcgtcgaCCTCAGTCCCTGCCATT | SalI |
| I-3cr | 2,401 | - | ggatccGCAACCCCAGAGGAGGAG | BamHI |
| I-3df | 2,402 | + | gcgtcgACCAGGAGGGGAACTTCT | SalI |
| I-3dr | 2,555 | - | cgggatccATAGATGCTGTCAGCTTC | BamHI |
| I-3ef | 2,556 | + | cgtcgaCCTCCAACCCATGCCCACT | SalI |
| I-3r | 2,720 | - | cgggatccTGGGGGAGCCTGTCT | BamHI |
| I-4f | 2,720 | + | gcgtcgACCCCATGTCCCCTGC | SalI |
| I-4r | 3,527 | - | cgggatccACAATGGCATGGAGC | BamHI |
| I-5f | 3,527 | + | gcgtcgacTCCTCCGCCAAGAC | SalI |
| I-5r | 4,319 | - | cggcagatctTTAGTTAAGAACCATGTCTC | BglII |
| I-6f | 4,317 | + | gcgtcgacTAAAGATCTGTATCCATTAGGAATG | SalI |
| I-6r | 4,868 | - | cgtGGATCCATTCCACAAAAACTCACAC | BamHI |
| Int3 | 4,868^5^ | - | CTCTGATAAAAGCCGTGGATCCATTCCAC | BamHI |

^1^- 5’ end of primer, relative to the liver-specific glucokinase mRNA start site.

^2^- Stand primer designed to.

^3^- Sequence from 5’ to 3’ with bases added to aid in cloning shown in lower case and restriction endonuclease cleavage sites underlined.

^4^- Restriction endonuclease cleavage sites used for subcloning.

^5^- Location identified the 3’ end of the BamHI restriction endonuclease cleavage site in the genomic sequence and included in reporter constructs
