# Supplementary material for: Expression of the Human Glucokinase Gene: Important Roles of the 5′ Flanking and Intron 1 Sequences
Source: PLoS One. 2012 Sep 20;7(9):e45824. doi: 10.1371/journal.pone.0045824 (PMC3447760; doi:10.1371/journal.pone.0045824)
Supplement: Table S2 — Primers used for the RT-PCR analysis of gene expression in HepG2 and L-02 cell lines. (DOCX) [file pone.0045824.s005.docx]

**Table S2. Primers used for the RT-PCR analysis of gene expression in HepG2 and L-02 cell lines.**

| Gene | Strand | Sequence | Product size | Annealing temp |
| --- | --- | --- | --- | --- |
| liver GCK | + | GCCTTGACTCTGGTAGAGCAG | 227 | 56 |
|  | - | ATCACCCTGAAGTTAGTGCCA |  |  |
| GCKR | + | GTTGGACCTTCGGATTAGCA | 379 | 55 |
|  | - | CCCAGAAACATGGGTTCACT |  |  |
| INSR | + | AGAGGCAGGCGGAAGACAGT | 482 | 62 |
|  | - | GATGCGATAGCCCGTGAAGT |  |  |
| IRS1 | + | AGTCTGGCTACTTGTCTGGC | 550 | 60.7 |
|  | - | ATCACTTTGGCACTCTGGTT |  |  |
| IRS2 | + | GGCTTGGTCGGTTGTCCTG | 507 | 70.6 |
|  | - | GCCTTGTTGGTGCCTCATCTAA |  |  |
| ChREBP | + | CGAGGTGGTGATGCGGGAATA | 406 | 64 |
|  | - | TGGGAGGCGGGAGTTGGTA |  |  |
| PKLR | + | TTCGTTTGTGAGCCTGACTG | 569 | 61 |
|  | - | TCACCAGGTTGTTGACATTGTA |  |  |
| G6PC | + | AAAGATAAAGCCGACCTACAGA | 494 | 60 |
|  | - | GGACGAGGGAGGCTACAATA |  |  |
| PCK1 | + | GAAATCTTAGCATGCCTCCA | 217 | 60 |
|  | - | AAATATCACACAGACACATGTGC |  |  |
| PYGL | + | TTCGGTATGAATATGGGATT | 460 | 56.7 |
|  | - | TTGAAACGGCGGATGA |  |  |
| GYS2 | + | CAACAACCGCACAGATAG | 536 | 63.2 |
|  | - | AAGGTGGTACTGAGGAAGG |  |  |
| FASN | + | TTCGTTTGTGAGCCTGACTG | 569 | 61 |
|  | - | TCACCAGGTTGTTGACATTGTA |  |  |
| SREBF | + | GCGGAGCCATGGATTGCAC | 311 | 64 |
|  | - | CTCTTCCTTGATACCAGGCCC |  |  |
| HNF4A | + | GCGTGGTGGACAAAGACAA | 459 | 62 |
|  | - | AGTGCCGAGGGACAATGTAG |  |  |
| PPARG | + | AGGAGCAGAGCAAAGAGGT | 475 | 62 |
|  | - | GAGGACTCAGGGTGGTTCA |  |  |
| FOXO1 | + | GATAAGGGTGACAGCAACAG | 496 | 60 |
|  | - | CAGACAGACTGGGTAAAGTAGAG |  |  |
| beta-Actin | + | CTGGGACGACATGGAGAAAA | 564 | 64 |
|  | - | AAGGAAGGCTGGAAGAGTGC |  |  |
| GAPDH | + | AGGTCGGAGTCAACGGATTTG | 532 | 62 |
|  | - | GTGATGGCATGGACTGTGGT |  |  |
