# Supplementary material for: Expression of the Human Glucokinase Gene: Important Roles of the 5′ Flanking and Intron 1 Sequences
Source: PLoS One. 2012 Sep 20;7(9):e45824. doi: 10.1371/journal.pone.0045824 (PMC3447760; doi:10.1371/journal.pone.0045824)
Supplement: Table S3 — Sizes of introns (in bp) in mammalian glucokinase genes. (DOCX) [file pone.0045824.s006.docx]

**TABLE S3. Sizes of introns (in bp) in mammalian glucokinase genes.**

|  | Intron | | | | | | | | | |
| --- | --- | --- | --- | --- | --- | --- | --- | --- | --- | --- |
| Species | Beta 1^1^ | Liver 1^1^ | 2 | 3 | 4 | 5 | 6 | 7 | 8 | 9 |
| Human | 35,445 | 5,609 | 875 | 1,195 | 891 | 109 | 1,926 | 1,031 | 732 | 216 |
| Chimpanzee | ?^2^ | 4,609 | 876 | 1,195 | 891 | 109 | 1,929 | 1,031 | 736 | 216 |
| Gorilla | 35,710 | 5,611 | 878 | 1,193 | 891 | 109 | 1,933 | 1,031 | 756 | 216 |
| Orangutan | 35,196 | 5,601 | 884 | 1,188 | 889 | 109 | 1,920 | 1,029 | 820 | 213 |
| Gibbon | 35,561 | 5,138 | 973 | 1,170 | 889 | 110 | 1,905 | 1,030 | 637 | 212 |
| Baboon | ? | 5,613 | 1,168 | 1,190 | 892 | 109 | 1,866 | 1,027 | 694 | 218 |
| Macaque | 36,380 | 5,649 | 1,164 | 1,190 | 894 | 109 | 1,863 | 1,028 | 647 | 215 |
| Marmoset | 30,533 | 5,533 | 866 | 1,099 | 873 | 107 | 1,928 | 1,020 | 745 | 226 |
| Tarsier | 5,211 | ? | 573 | 1,507 | 990 | 110 | 2,117 | 1,185 | ? | ? |
| Bushbaby | ? | 7,111 | 548 | 1,054 | 1,209 | 108 | 1,065 | 948 | 630 | 253 |
| Mouse lemur | ? | 5,666 | 525 | 1,090 | 900 | 95 | 2,195 | 656 | 708 | 231 |
| Mouse | 38,530 | 3,942 | 429 | 1,075 | 2,304 | 106 | 1,932 | 1,105 | 781 | 270 |
| Rat | 31,458 | 4,657 | 403 | 966 | 2,386 | 107 | 1,985 | 1,133 | 776 | 467 |
| Chinese hamster | 28,938 | 5,314 | 474 | 1,078 | 1,265 | 105 | 2,505 | 1,303 | 240 | 222 |
| Kangaroo rat | 21,601 | ? | ? | ? | ? | ? | 1,675 | 1,345 | 439 | 114 |
| Guinea pig | 30,259 | 2,482 | 391 | 886 | 844 | 299 | 1,772 | ? | ? | 77 |
| Squirrel | ? | 5,311 | 526 | 1,060 | 1,146 | 111 | 2,392 | 798 | 486 | 249 |
| Pika | ? | 3,091 | 442 | 596 | 770 | 72 | 1,263 | ? | ? | 128 |
| Rabbit | 30,095 | 3,638 | 453 | 678 | 940 | 77 | ? | ? | ? | 91 |
| Cow | 26,237 | 3,949 | 545 | 1,074 | 1,589 | 100 | 1,988 | 918 | 633 | 255 |
| Sheep | 26,197 | ? | 513 | 1,092 | 1,563 | 202 | ? | ? | ? | ? |
| Dolphin | 19,651 | 4,100 | 580 | 1,016 | 1,670 | 188 | 2,611 | 1,195 | 750 | 257 |
| Pig | 33,915 | 4,323 | 815 | 1,040 | 2,148 | 201 | 1,856 | 1,314 | 679 | 180 |
| Alpaca | ? | ? | ? | ? | ? | ? | ? | ? | ? | ? |
| Horse | 41,533 | 4,932 | 559 | 1,099 | 1,229 | 192 | 1,483 | 1,000 | 614 | 309 |
| Dog | 33,688 | 3,850 | 432 | 926 | 990 | 135 | 1,979 | ? | ? | ? |
| Cat | 35,738 | ? | 1,002 | 3,668 | 963 | 132 | 2,177 | 900 | 521 | 274 |
| Ferret | 28,028 | 3,833 | 150 | 963 | 1,054 | 155 | 1,424 | 825 | 442 | 179 |
| Panda | 30,508 | 3,788 | 526 | 1,009 | 1,113 | 155 | 1,659 | 858 | 508 | 273 |
| Little brown bat | 6,952 | ? | 430 | 353 | 819 | 172 | 918 | 717 | 271 | 278 |
| Flying fox bat | ~8,700^3^ | ? | 407 | 785 | 1,249 | 164 | 640 | 711 | 312 | 222 |
| Tree shrew | ? | 3,570 | ? | ? | ? | 94 | ? | ? | ? | 188 |
| Hedgehog | 32,625 | 3,565 | 465 | 1,801 | 2,389 | 146 | 1,436 | 100 | 369 | 741 |
| Shrew | ? | ? | ? | ? | ? | 173 | 2,176 | 808 | 446 | 76 |
| Elephant | 127,804 | 7,987 | 1,446 | 1,104 | 586 | 180 | 2,372 | 785 | 715 | 414 |
| Hyrax | 16,226 | 7,649 | 892 | 1,082 | 571 | 195 | 3,401 | 955 | 468 | 393 |
| Tenrec | ? | ? | ? | ? | 480 | 220 | 2,530 | 1,197 | 390 | 1,787 |
| Armadillo | 22,461 | ? | 527 | 321 | 938 | 125 | 1,861 | 997 | 208 | 709 |
| Sloth | 4,639 | ? | 616 | 100 | 1,689 | 158 | 1,696 | 1,470 | 3,749 | 360 |
| Opossum | 66,370 | ? | 2,533 | 1,022 | 791 | 294 | 5,412 | 1,873 | 1,027 | 2,892 |
| Wallaby | ? | ? | ? | 102 | 733 | 325 | 2,494 | 1,839 | 943 | 3,572 |
| Tasmanian devil | 63,871 | 8,570 | 2,198 | 1,014 | 1,848 | 328 | 3,084 | 1,913 | 1,153 | 3,096 |
| Platypus | 9,815 | ? | 283 | 399 | 84 | 567 | 598 | 1,665 | 261 | 92 |

^1^- Distance from 3’ end of beta-cell and liver specific exons to the 5’ end of exon 2.

^2^- ?, indicates that the size of the intron is unknown, which can be due to gaps in, or problems with, the genomic assembly.

^3^- Coding region of exon 1 is missing but the 5’ untranslated sequence is present.
