# Supplementary material for: Expression of the Human Glucokinase Gene: Important Roles of the 5′ Flanking and Intron 1 Sequences
Source: PLoS One. 2012 Sep 20;7(9):e45824. doi: 10.1371/journal.pone.0045824 (PMC3447760; doi:10.1371/journal.pone.0045824)
Supplement: Table S4 — Number and amount of repetitive DNA elements located between the beta-cell and liver-specific exons of mammalian glucokinase gene. (DOCX) [file pone.0045824.s007.docx]

**Table S4. Number and amount of repetitive DNA elements located between the beta-cell and liver-specific exons of mammalian glucokinase gene.**

|  |  | Number of repetitive elements | | | |  |
| --- | --- | --- | --- | --- | --- | --- |
| Species | Length | SINEs | LINEs | LTR | Other | % Rep^1^ |
| Human | 29626 | 14 | 10 | 10 | 6 | 60.7 |
| Marmoset | 24797 | 10 | 5 | 9 | 4 | 56.7 |
| Mouse | 34421 | 21 | 4 | 16 | 4 | 59.2 |
| Rat | 26629 | 35 | 2 | 15 | 3 | 41.2 |
| Guinea pig | 27626 | 35 | 7 | 7 | 1 | 62.8 |
| Rabbit | 26525 | 21 | 7 | 6 | 1 | 38.5 |
| Cow | 22081 | 16 | 8 | 5 | 5 | 37.5 |
| Pig | 29383 | 18 | 11 | 9 | 2 | 49.4 |
| Horse | 36391 | 12 | 11 | 12 | 5 | 50.3 |
| Dog | 29629 | 10 | 6 | 7 | 9 | 31.4 |
| Panda | 26509 | 14 | 6 | 8 | 4 | 40.6 |
| Hyrax | 8372 | 2 | 5 | 3 | 1 | 51.8 |
| Tasmanian devil | 55078 | 52 | 54 | 4 | 12 | 49.7 |

^1^- Parentage of the sequence that is made of repetitive elements.
